# Decreased Fatty Acid Oxidation Gene Expression in Pre-Eclampsia According to the Onset and Presence of Intrauterine Growth Restriction

**DOI:** 10.3390/nu15183877

**Published:** 2023-09-06

**Authors:** Alejandra Abascal-Saiz, Eva Fuente-Luelmo, María Haro, Victoria Fioravantti, Eugenia Antolín, María P. Ramos-Álvarez, José L. Bartha

**Affiliations:** 1 Department of Obstetrics and Gynecology, Division of Maternal and Fetal Medicine, Institute for Health Research—IdiPAZ (La Paz University Hospital—Universidad Autónoma de Madrid), Paseo de la Castellana 261, 28046 Madrid, Spain; alejandra_as@hotmail.com (A.A.-S.); eantolin@salud.madrid.org (E.A.); 2 Department of Biochemistry and Molecular Biology, Faculty of Pharmacy, CEU-San Pablo University, 28668 Boadilla del Monte, Madrid, Spain; eva.defuenteluelmo@ceu.es (E.F.-L.); maria.harogarcia@ceu.es (M.H.); pramos@ceu.es (M.P.R.-Á.); 3 General Pediatrics, Sanchinarro Health Center, 28050 Madrid, Spain; v.fioravantti@gmail.com

**Keywords:** pre-eclampsia, fatty acid oxidation, long-chain 3-hydroxyacyl-CoA dehydrogenase LCHAD, medium-chain acyl-CoA dehydrogenase MCAD, carnitine palmitoyltransferase CPT, gene expression, qPCR, lipidomic, placental villous explants, placental function

## Abstract

Mitochondrial fatty acid oxidation (FAO) is lower in placentas with pre-eclampsia. The aim of our study was to compare the placental mRNA expression of FAO enzymes in healthy pregnancies vs. different subgroups of pre-eclampsia according to the severity, time of onset, and the presence of intrauterine growth restriction (IUGR). By using real-time qPCR, we measured the mRNA levels of long-chain 3-hydroxyacyl-CoA dehydrogenase (LCHAD), medium-chain acyl-CoA dehydrogenase (MCAD), and carnitine palmitoyltransferases 1A and 2 (CPT1A, CPT2) on the maternal side (anchoring villi in the basal decidua) and on the fetal side (chorionic plate) of the placenta (*n* = 56). When compared to the controls, LCHAD, MCAD, and CPT2 mRNA had decreased in all pre-eclampsia subgroups globally and on the fetal side. On the maternal side, LCHAD mRNA was also lower in all pre-eclampsia subgroups; however, MCAD and CPT2 mRNA were only reduced in severe and early-onset disease, as well as CPT2 in IUGR (*p* < 0.05). There were no differences in CPT1A mRNA expression. We conclude that the FAO enzymes mRNA in the placenta was lower in pre-eclampsia, with higher reductions observed in severe, early-onset, and IUGR cases and more striking reductions on the fetal side.

## 1. Introduction

### 1.1. Etiopathogenesis of Pre-Eclampsia

Pre-eclampsia is a prevalent complex multisystemic disease that affects between 1 and 5.6% of pregnancies globally. It is associated with a high maternal–fetal morbidity and mortality rate around the world and is considered the second most common cause of maternal deaths worldwide, only behind hemorrhage [1]. Pre-eclampsia is defined by diagnostic criteria as new-onset hypertension after >20 weeks of gestation and at least one of the following: proteinuria, maternal organ dysfunction (renal, hepatic, neurological, or hematological), or uteroplacental dysfunction (e.g., placental abruption, angiogenic imbalance, intrauterine growth restriction (IUGR), or intrauterine fetal death) [2,3].

The pathogenic basis of pre-eclampsia is still not well established, and no curative treatment has yet been developed. The accepted patho-physiological hypothesis for pre-eclampsia is the failure of trophoblastic invasion with a subsequent placental defect and uteroplacental malperfusion, although oxidative stress and inflammatory and metabolic changes also appear to be involved [4]. The placenta is an organ with great adaptive capacity throughout pregnancy. It remains the main focus of research in pre-eclampsia, as greater knowledge of placental function might help develop strategies for the prevention and treatment of pre-eclampsia, as well as provide a new approach for future placental studies. In the last decade, the “omic” sciences have described genomic, transcriptomic, proteomic, and metabolomic features in the placenta of patients with pre-eclampsia to study its etiopathogenesis and, thus, to search for predictive markers and therapeutic targets [5,6]. Indeed, in recent years, important advances have been made in the prediction and prevention of preterm pre-eclampsia [1,4,7]. Pre-eclampsia has been subgrouped by gestational age at presentation in early-onset (EOPE, at <34 weeks of pregnancy) or late-onset (LOPE, ≥34 weeks) [1], and more and more studies are showing that they behave as two different conditions. In EOPE, the pathogenesis is more related to placental insufficiency and damage, leading to a clinical situation similar to atherosclerosis during gestation, whereas, in LOPE, there is a metabolic imbalance that leads to an insufficient supply in terms of fetal demands [8]. Therefore, the study of lipids in the maternal serum and placenta in the different types of pre-eclampsia is of great importance as an alternative to other pathogenic routes [9].

### 1.2. Placental Lipid Metabolism

In the first half of gestation, the maternal metabolism is fundamentally anabolic, but in the second half of the process of lipid utilization, this is reversed, moving towards a catabolic state with the degradation of fatty acids in the mitochondria through β-oxidation (fatty acid oxidation, FAO) in favor of a greater transfer towards the placenta and fetus [10,11]. Beta-oxidation degrades fatty acids by various enzymatic chain reactions that are fatty acid chain-length-dependent, and this is mediated by enzymes such as medium-chain acyl-CoA dehydrogenase (MCAD) or long-chain 3-hydroxyacyl-CoA dehydrogenase (LCHAD). Short- and medium-chain fatty acids (SC/MC-FA) do not require transporters and diffuse directly into the mitochondrial matrix. Carnitine palmitoyltransferases 1 and 2 (CPT1 and CPT2) are present on the outer and inner mitochondrial membrane, respectively, and together enable long-chain fatty acids (LC-FA) to be transported as acylcarnitines from the cytosol into the mitochondrial matrix, for their utilization in FAO later. CPT1 is the main regulator of FAO and has three isoforms (CPT1A, CPT1B, and CPT1C) that are expressed in most tissues at differing isoform ratios depending on the tissue type, although their specific characteristics remain under investigation [12,13]. CPT1A is the isoform with the highest gene expression in the placenta [14].

As previously described by our group, LCHAD gene expression and FAO activity are reduced in pre-eclampsia when compared to controls [15]. Therefore, placental FAO disorders might play a role in the pathophysiology of pre-eclampsia. In addition, we have previously demonstrated that in healthy pregnant women, the maternal side of the placenta is more active in the esterification of fatty acids, whereas lipid storage concentrates more on the fetal side [16]. 

### 1.3. Purpose of the Study

Our current study focuses on an ex vivo analysis of the placental gene expression of LCHAD, MCAD, CPT1A, and CPT2 from patients with pre-eclampsia. The main aim was to compare healthy pregnant women to patients with different degrees of severity of pre-eclampsia, different times of onset, and an association with IUGR. We also analyzed the difference in FAO enzyme mRNA expression between the maternal and fetal sides of the placenta in the different subgroups of patients.

## 2. Materials and Methods

### 2.1. Study Participants

A prospective observational study was carried out in a tertiary university hospital (Department of Obstetrics and Gynecology) for two years. This study was approved by the Local Ethics Committee, and all the participants signed informed consent forms.

**Figure 1:**
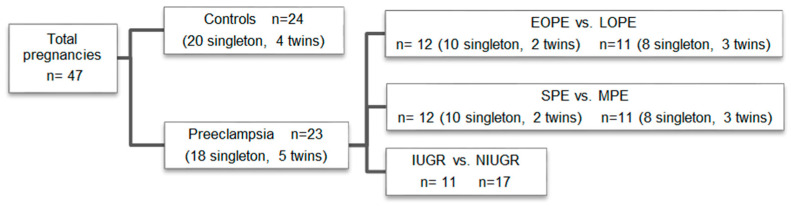
Study groups. *Key*: EOPE: early-onset pre-eclampsia, LOPE: late-onset pre-eclampsia, SPE: severe pre-eclampsia, MPE: mild pre-eclampsia, IUGR: intrauterine growth restriction, NIUGR: not intrauterine growth restriction. The term “twins” is in reference to the number of twin pregnancies in each group.

Forty-seven women were included and allocated into two groups, with 24 healthy patients as a control group and 23 with pre-eclampsia. The pre-eclampsia group was subdivided according to time of onset: early or late (EOPE vs. LOPE), according to severity: severe or mild (SPE vs. MPE), and according to the presence of IUGR (IUGR vs. NIUGR). The study groups are shown in Figure 1. In the case of twin pregnancies, the placentas were analyzed independently. The study was finally performed on 56 placentas. To avoid the potential effects of labor contractions on placental metabolism, all deliveries were performed by cesarean section due to clinical reasons not affecting placental metabolism or perfusion, as shown in Table 1.

The inclusion criteria for the healthy control subjects were as follows: normal blood pressure during pregnancy, pregnancy at term, no medical history of chronic metabolic diseases or any pathology that could involve lipid or carbohydrate metabolism disorders, and no complications during pregnancy. The exclusion criteria for the control and pre-eclampsia groups included major fetal anomalies, women with a history of inborn FAO disorder, acute fatty liver of pregnancy (AFLP), and pre-existing or gestational diabetes. The diagnosis and severity classification of pre-eclampsia was made using the criteria provided by the International Society for the Study of Hypertension in Pregnancy (ISSHP) [3]. Severe pre-eclampsia (SPE) is characterized by the onset of severe hypertension and/or any signs or symptoms of significant end-organ dysfunction, such as central nervous system dysfunction, liver involvement, hematological complications, renal insufficiency, or pulmonary edema [3]. HELLP syndrome (hemolysis, elevated liver enzymes, low platelets), diagnosed using the Tennessee classification [17], is also considered a subtype of SPE. Those patients who did not meet the severity criteria were considered mild pre-eclampsia (MPE). The diagnosis of IUGR was made via ultrasound and following the ISUOG Practice Guidelines [2]; fetuses without the IUGR criterion were referred to as “not intrauterine growth restriction” (NIUGR). 

### 2.2. Sample Collection

The fresh whole placenta was transported on dry ice to the laboratory within 2 h of delivery. Six 100 mg cotyledon fragments (chorionic villous explants) were collected from each placenta by removing the decidual tissue, calcium deposits, and large vessels from those regions near the chorionic plate and the basal plate. In both groups, explants were taken from the central, intermediate, and peripheral locations to the umbilical cord insertion. Three 100 mg explants were obtained close to the chorionic plate (hereinafter referred to as “fetal side”), and another three 100 mg explants were obtained close to the anchoring villi into the decidua basalis (referred to as “maternal side”). They were washed in 500 μL of cold phosphate-buffered saline (PBS). The global results of each assay were obtained by calculating the mean of the maternal and fetal sides of the placenta.

### 2.3. Quantification of Placental mRNA Levels

Total RNA was extracted from 25–30 mg placental tissues using QIAzol Lysis Reagent/Chloroform and the RNeasy Mini Kit (Qiagen, Venlo, The Netherlands), following the manufacturer’s protocol, including DNase treatment to remove genomic DNA contamination. RNA quantity and purity were assessed by spectrophotometry by measuring the OD260, OD260/280 ratio, and OD260/230 by using a NanoVue Plus (GE Healthcare, Chicago, IL, USA). Three µg cDNA synthesis was carried out using random hexamer primers and the components from the RevertAid H Minus First Strand cDNA Synthesis Kit (Thermo Scientific, Waltham, MA, USA). Primers were designed, and their specificity was determined using Primer-BLAST tool (Supplementary Table S1) [18]. Primer specificity was confirmed by agarose gel electrophoresis (2%) after real-time quantitative PCR (RT-qPCR) reaction. The concentration of cDNA in each PCR was 1 ng/µL. Standard curves for each target were prepared from the pooled samples of first-strand cDNA. We analyzed Glyceraldehyde-3-phosphate dehydrogenase (GAPDH), Ribosomal Protein L30 (RPL30), LCHAD, MCAD, CPT1A, and CPT2 by using the RT-qPCR reaction performed with cDNA using Quantimix Easy Kit (Biotools, South San Francisco, CA, USA). Reactions were run on a CFX96 Real-Time PCR Detection System (Bio-Rad, Hercules, CA, USA). The cycle conditions were as follows: 9 min at 95 °C for enzyme activation, 40 cycles of denaturation at 95 °C for 10 s, specific annealing temperature for each case, extension at 72 °C for 45 s, followed by a melting curve ranging from 65 °C to 95 °C by steps of 0.5 °C for 5 s. Two technical replicates of qPCR were performed per sample. Relative mRNA levels were calculated by using the Livak method [19]. The LCHAD, MCAD, CPT1A, and CPT2 mRNA levels were normalized by using the GAPDH and RPL30 housekeeping genes [20]. The use of GAPDH as a constitutive gene in human placenta is controversial, so a second constitutive housekeeping gene was included in the study: RPL30 [21,22]. The raw data of GAPDH and RPL30 were analyzed for all samples, proving no differences in their mRNA levels between the groups of the study. The fold change in mRNA was calculated relative to the value of the maternal side of healthy pregnancies. The global analysis of the placenta was calculated by using the mean of the quantification cycle (Cq) value of both placental sides; then, the fold change in this value was performed using placentas from healthy pregnancies as a control. Data were expressed in arbitrary units (a.u.).

### 2.4. Statistical Analysis

All data were analyzed using SPSS 20.0. The normal distribution of variables was assessed by the Kolmogorov–Smirnov test. The Student’s *t*-test and ANOVA were used for the variables with a normal independent distribution, and the paired *t*-test was used for the paired samples (data were expressed as mean ± standard deviation). The variables with an independent, non-normal distribution were analyzed by using the Mann–Whitney U-test and Kruskal–Wallis test, and the paired samples by using the Wilcoxon test (data are shown as the median and interquartile range). Differences were considered significant at a *p*-value of <0.05.

## 3. Results

### 3.1. Maternal, Obstetric, and Perinatal Outcomes

The maternal characteristics and obstetric–perinatal outcomes are shown in Table 1. The demographic characteristics of both groups did not show significant differences. We observed a lower gestational age at study, neonatal, and placental weights in the EOPE group, followed by LOPE, when compared to uncomplicated pregnancies. There was a higher incidence of IUGR in the pre-eclampsia groups, especially in EOPE. 

### 3.2. Analysis of the Placental mRNA Levels

#### 3.2.1. Comparison between Pre-Eclampsia and Controls

- LCHAD

**Figure 2:**
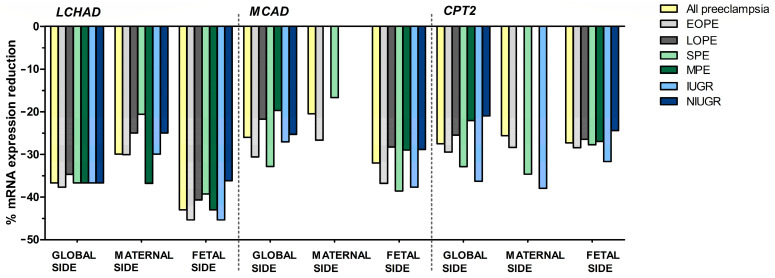
Percentage of mRNA expression reduction in pre-eclampsia subgroups vs. controls. Only statistically significant (*p* < 0.05) percent reductions (compared to controls) are shown. *Key:* LCHAD: long-chain 3-hydroxyacyl-CoA dehydrogenase, MCAD: medium-chain acyl-CoA dehydrogenase, CPT2: carnitine palmitoyltransferase, EOPE: early-onset pre-eclampsia, LOPE: late-onset pre-eclampsia, SPE: severe pre-eclampsia, MPE: mild pre-eclampsia, IUGR: intrauterine growth restriction, NIUGR: not intrauterine growth restriction.

**Figure 3:**
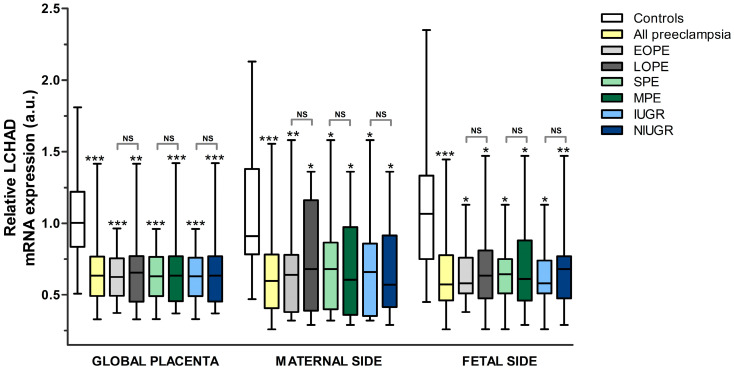
Relative LCHAD mRNA expression (a.u.). All pre-eclampsia subgroups were compared to the controls (statistical significance immediately above the upper whisker for each group). Additionally, the pre-eclampsia subgroups were compared according to time of onset, severity, or association with IUGR (statistical significance above brackets, indicating groups compared). Statistical significance: NS: not significant, * *p* ≤ 0.05, ** *p* ≤ 0.01, *** *p* ≤ 0.001. *Key:* LCHAD: long-chain 3-hydroxyacyl-CoA dehydrogenase, EOPE: early-onset pre-eclampsia, LOPE: late-onset pre-eclampsia, SPE: severe pre-eclampsia, MPE: mild pre-eclampsia, IUGR: intrauterine growth restriction, NIUGR: not intrauterine growth restriction.

LCHAD mRNA expression in the placenta was found to be lower in the overall pre-eclampsia sample vs. controls both globally and when studying the maternal and fetal sides separately (*p* = 1.1 × 10^−5^, 5.6 × 10^−4^, and 5.5 × 10^−4^, respectively) and independently of the time of onset, severity, or the association with IUGR in pre-eclampsia (Figure 2 and Figure 3, Supplementary Table S2). Globally, LCHAD mRNA reduction was similar in all pre-eclampsia subgroups when compared to the controls (−37.7% in EOPE, −34.7% in LOPE, and −36.7% in the rest of the subgroups). On the maternal side, higher reductions were observed in MPE when compared to uncomplicated pregnancies (−36.8%), followed by EOPE, IUGR pre-eclampsia, and total pre-eclampsia (−30.0% in all of them). The more striking reductions in LCHAD mRNA expression, when compared to the controls, were found in the analysis of the fetal side, with a 45.4% decrease in the EOPE and IUGR cases, followed by −43.0% in MPE and in the overall pre-eclampsia sample, −40.7% in LOPE, −39.3% in SPE, and −36.2% in NIUGR.

- MCAD

**Figure 4:**
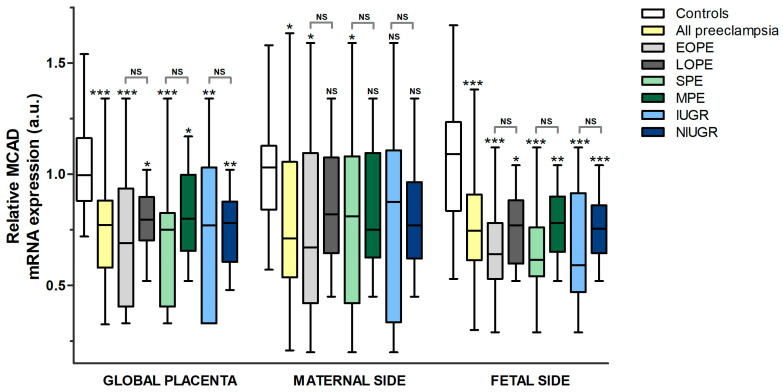
Relative MCAD mRNA expression (a.u.). All pre-eclampsia subgroups were compared to the controls (statistical significance immediately above the upper whisker for each group). Additionally, pre-eclampsia subgroups were compared according to time of onset, severity, or association with IUGR (statistical significance above brackets, indicating groups compared). Statistical significance: NS: not significant, * *p* ≤ 0.05, ** *p* ≤ 0.01, *** *p* ≤ 0.001. *Key:* MCAD: medium-chain acyl-CoA dehydrogenase, EOPE: early-onset pre-eclampsia, LOPE: late-onset pre-eclampsia, SPE: severe pre-eclampsia, MPE: mild pre-eclampsia, IUGR: intrauterine growth restriction, NIUGR: not intrauterine growth restriction.

In general terms, the reduction in MCAD gene expression at the placenta in pre-eclampsia vs. the controls was lower than in LCHAD (Figure 2 and Figure 4, Supplementary Table S3). Globally, MCAD mRNA expression at the placenta was found to be lower (*p* < 0.05) in all subgroups of pre-eclampsia vs. healthy pregnancies. The most important differences were in SPE (−32.8%) and EOPE (−30.6%), followed by IUGR, the overall pre-eclampsia sample, and NIUGR (−27.1%, −26%, and −25.3%, respectively). On the fetal side of the placenta, similar to what occurs in the global placenta, MCAD mRNA expression was reduced in all pre-eclampsia groups when compared to the controls (*p* < 0.05), and the largest decreases were found in the following subgroups: SPE (−38.6%), IUGR (−37.7%), EOPE (−36.8%), and all pre-eclampsia (−32.0%). The decrease in gene expression on the fetal side was smaller and similar in the MPE, NIUGR, and LOPE subgroups (−28.9% in all of them) when compared to the controls. In contrast, on the maternal side of the placenta, a significant decrease in MCAD expression was found only in the overall pre-eclampsia sample (−20.5%), EOPE (−26.7%), and SPE (−16.7%) groups when compared to the uncomplicated pregnancies with no differences vs. the LOPE, MPE, IUGR, and NIUGR subgroups.

- CPT1A

No statistically significant differences were obtained when comparing CPT1A mRNA expression between the controls and pre-eclampsia, nor between the controls and the different subgroups of pre-eclampsia, both in global terms and on the maternal or fetal side of the placenta (Supplementary Table S4).

- CPT2

**Figure 5:**
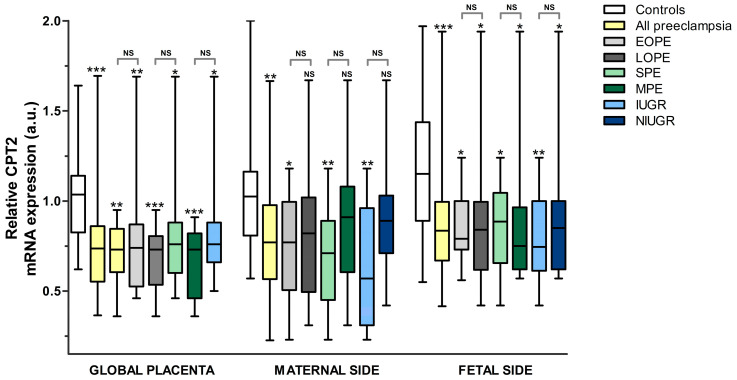
Relative CPT2 mRNA expression (a.u.). All pre-eclampsia subgroups were compared to the controls (statistical significance immediately above the upper whisker for each group). Additionally, pre-eclampsia subgroups were compared according to time of onset, severity, or association with IUGR (statistical significance above brackets, indicating groups compared). Statistical significance: NS: not significant, * *p* ≤ 0.05, ** *p* ≤ 0.01, *** *p* ≤ 0.001. *Key:* CPT2: carnitine palmitoyltransferase, EOPE: early-onset pre-eclampsia, LOPE: late-onset pre-eclampsia, SPE: severe pre-eclampsia, MPE: mild pre-eclampsia, IUGR: intrauterine growth restriction, NIUGR: not intrauterine growth restriction.

As was the case for MCAD, CPT2 mRNA expression at the placenta was found to be lower (*p* < 0.05) in all subgroups of pre-eclampsia vs. the controls globally and on the fetal side but not in all the subgroups of pre-eclampsia on the maternal side (Figure 2 and Figure 5, Supplementary Table S5). Globally, the greatest differences were in IUGR (−36.3%), SPE (−32.9%), EOPE (−29.5%), the overall pre-eclampsia sample (−27.5%), and LOPE (−25.5%). On the fetal side of the placenta, the most important difference, when compared to uncomplicated pregnancies, was a 31.7% reduction in the IUGR group. On the maternal side of the placenta, CPT2 only had a significant decrease in all pre-eclampsia (−25.6%), EOPE (−28.4%), SPE (−34.6%), and IUGR (−38.0%) groups when compared to the controls.

#### 3.2.2. Comparison between the Different Subgroups of Pre-Eclampsia

When analyzing the pre-eclampsia subgroups separately (EOPE vs. LOPE, MPE vs. SPE, and IUGR vs. NIUGR), both globally and on each placental side, no significant differences in placental LCHAD, MCAD, CPT1A, and CPT2 mRNA expression were found (Supplementary Tables S2–S5).

#### 3.2.3. Comparison between Sides of the Placenta in Each Subgroup

LCHAD, MCAD, CPT1A, and CPT2 gene expression were compared between the maternal and fetal sides of the placenta within each group included in the study (for example, the different sides of the placenta only in the EOPE group), but these differences did not reach statistical significance (Supplementary Tables S2–S5).

## 4. Discussion

### 4.1. Main Findings

This is the first study to analyze the gene expression of FAO enzymes at the maternal and fetal placental sides in pre-eclampsia and compare these to controls; it is also the first to analyze across different subtypes of pre-eclampsia according to the time of onset, severity, or association with IUGR. The main findings demonstrate lower LCHAD, MCAD, and CPT2 gene expression in the placenta in pre-eclampsia, both at the chorionic and basal plates. The most important reductions in mRNA expression from these genes are related to EOPE, SPE, and pre-eclampsia with IUGR. No differences were found in CPT1A mRNA expression between pre-eclampsia and the controls.

### 4.2. Interpretation of Results

The importance of an adequate fatty acid metabolism during pregnancy is undeniable. Fatty acids are an essential substrate for energy supply, as they are the precursors of acetyl-CoA and ketone bodies. The severe clinical consequences described in the most frequent inborn FAO disorders (MCAD, LCHAD, or very-long-chain acyl-coA dehydrogenase (VLCAD) deficiencies) warn us of their importance in postnatal life [23,24]; however, adequate FAO is also required earlier for fetal and placental energy support. Although the finding of fetal malformations is exceptional in FAO congenital disorders, in the case of CPT2 or multiple acyl-coA dehydrogenase (MADD) deficiencies, cerebral anomalies, or polycystic kidneys have been described [25]. However, fetal FAO defects are frequently associated with IUGR, preterm delivery, fetal morbidity, and intrauterine death [26,27,28]. In addition, pregnant women carrying a fetus with an FAO disorder are at a higher risk for AFLP or HELLP syndrome (1:10) due to an accumulation of toxic metabolites in the placenta and maternal liver [29]. These findings are consistent with our study, in which we found that lower FAO enzymes gene expression in the placenta is associated with the most severe cases of pre-eclampsia or those with IUGR.

In the last two decades, it has been shown that FAO enzymes are even more active in the placenta than in the maternal liver [27,30], with higher gene expression in the placenta than in maternal adipose tissue or maternal umbilical cord blood [31]. With respect to fatty acid chain length, higher placental enzyme activity has been demonstrated in those involved in LC-FAO when compared to MC/SC-FAO, both in the first trimester and at term [30], yet with this activity being inversely proportional to gestational age during the second and third trimesters [10,27]. The gene expression and activity of different FAO enzymes (VLCAD, LCHAD, MCAD, SCHAD, and CPT1 or 2) are observed in various tissues in human embryos as early as five weeks of development, highlighting the key role of FAO in early pregnancy [26,32], with a higher expression in the syncytiotrophoblast than in the cytotrophoblast [27]. Physiologically, higher levels of LCHAD and MCAD mRNA are found in term placentas than in maternal or fetal blood, with a higher expression of LCHAD vs. MCAD in the placenta. LCHAD levels are similar in maternal and fetal blood. However, MCAD levels are higher in fetal blood [31]. In our study, we could not demonstrate FAO enzymes mRNA differences between placental sides. These findings are in agreement with our previous paper, in which [^3^H]-palmitate was used as a free fatty acid (FFA) tracer to measure placental FAO by a vapor-phase chromatography assay, and no differences were observed either when comparing the maternal and fetal sides of the placenta or between singleton and twin pregnancies in healthy pregnant women at term [16].

In a different study from our working group with a smaller population, a decrease in FAO activity and the lower expression of LCHAD mRNA in placentas with pre-eclampsia was already described when compared to the controls, without reaching significant differences for MCAD [15]. Our current article adds value to the latter, as it reaffirms the decrease in LCHAD gene expression, and it also demonstrates the reduction in MCAD and CPT2 mRNA expression in the placentas of patients with pre-eclampsia. Moreover, when currently analyzing the expression according to the time of onset, we observed a greater reduction in EOPE than in LOPE when compared to the controls. In the placentas of healthy pregnant patients, the maximum FAO activity and levels are described between 12–28 weeks [27]. Therefore, although there is no evidence at the genomic level, a physiological reduction in healthy pregnancies with lower gestational age does not seem plausible, and decreased FAO enzymes mRNA expression might be attributed to pre-eclampsia. The results from various experiments carried out in pre-eclampsia-like mouse models [33,34], in vitro trophoblast cell cultures [35,36], or in placental explants [37,38] have corroborated this as well.

In the pathogenesis of pre-eclampsia, oxidative stress and inflammation are associated with lipid disorders. Dysfunction in β-oxidation enzymes in the placenta leads to the accumulation of toxic metabolic intermediates of fatty acid catabolism, promotes the production of lipid peroxides, and reduces the production of antioxidants in the placenta, releasing free radicals into the maternal circulation with subsequent endothelial dysfunction, excessive inflammation, and an increase in the production of antiangiogenic factors. On the other hand, the lipid metabolites accumulate in intracellular lipid deposits, leading to mitochondrial damage that accelerates apoptosis and decreases the invasive capacity of trophoblast. In pre-eclampsia, due to the inhibition of the mitochondrial respiratory chain, there is an increase in lipid peroxidation and nicotinamide adenine dinucleotide phosphate oxidase (NADPH) metabolism with an elevation of free radicals and a decrease in antioxidants secondary to, for example, alterations in glutathione metabolism [6,24,39]. Studies similar to ours that have compared the placental explants in controls vs. SPE demonstrate the downregulation of some proteins that participate in mitochondrial dysfunction by different pathways (fusion, autophagy, and biogenesis). This promotes lower FAO, resulting in lower placental energy production in pre-eclampsia and an accumulation of omega-3 and omega-6 fatty acids in mitochondria [40], acting as prostaglandin and leukotriene precursors, which leads to inflammation, vasoconstriction, endothelial damage, the inhibition of angiogenesis, and decreased platelet aggregation. Previous reports confirm a reduction in LCHAD mRNA and protein expression in SPE vs. controls, with a more marked reduction in early severe cases [35,36,38]. This reduction is corrected in serum-free trophoblast cells in vitro by adding NADPH and p38 mitogen-activated protein kinase (p38MAPK) inhibitors [35], the signaling pathway of which plays an essential role in regulating cellular processes, especially inflammation. Increased oxidative stress in pre-eclampsia and its relationship with FAO dysfunction has also been described in other studies in pre-eclampsia-like mouse models [34,39,41] and in EOPE placentas [37], showing a higher expression of P47phox NADPH subunit, p38MAPKα/ Nuclear factor-κB (NF-κB), and Cyclooxygenase-2 (COX-2). They all presented a positive correlation with serum FFA and a negative correlation with LCHAD mRNA and protein expression. All of these differences in gene expression were more remarkable in pre-eclampsia-like mouse models with worse lipid profiles, such as EOPE, a high-fat diet group, and ApoE-knockout mice (suffering hypercholesterolemia) [34,36].

In another recent study, we reported an increase in the concentration of total and protein-adjusted triglycerides on the fetal side of the placenta in healthy, full-term pregnant women. Oil Red-O histological staining supported this finding by evidencing higher lipid deposition on the fetal side [16]. Similarly, in our current analysis, the greatest reductions in FAO enzymes gene expression were obtained on the fetal side when comparing pre-eclampsia vs. controls, which would consequently lead to an increase in lipids at that site. We can assume that this might be due to a damaged placenta that requires its own energy supply, with worsening function on the fetal side. The highest percentages of reduction, even on the maternal side of the placenta, are observed in early or severe cases or those associated with IUGR, in which placental insufficiency is more evident.

In general, in our analysis, when compared to controls, the percentages of LCHAD gene expression reduction in pre-eclampsia are higher in relation to the reduction in MCAD. The selective use of PUFA by the placenta and the fetus with respect to MC-FFA in normal conditions could be the explanation for this difference [42]. However, all of the evidence above suggests that placentas with pre-eclampsia preferentially use the FFA that can be metabolized directly without extra modification and/or transportation steps, promoting a more efficient energy supply. Reports about the metabolome in placentas with pre-eclampsia describe a decrease in MC-FA and an accumulation of LC-FA, in addition to a reduction in *trans*-double-bonded FA and the retention of *cis*-double-bonded FFA (the latter needs extra isomerization to complete FAO) [43].

In our study and in agreement with others previously published [44], the CPT1A mRNA levels did not differ between pre-eclampsia and the controls. CPT1 mRNA concentration is normally elevated under conditions requiring an increase in LC-FAO, such as fasting or during a hyperlipidemic diet, due to a decrease in malonyl coA (its main inhibitor and a precursor of lipogenesis) [13]. The decreased expression of CPT2 and LCHAD mRNA in pre-eclampsia leads to the failure of LC-FAO and, subsequently, to a cytosolic LC-FA-rich environment that could lead to stable or even rising CPT1 concentrations. Recent in vitro studies have linked increased CPT1A expression in the placenta to the development of pre-eclampsia by inhibition of trophoblast cell invasion and migration [45]. Consistent with this theory, maternal and umbilical cord serum carnitine and acyl-carnitine levels increase in pre-eclampsia [46,47,48,49]. In addition, a maternal high-fat diet in rats downregulated the mRNA and protein expressions of CPT2 in the placenta [50]. Mitochondria in patients with pre-eclampsia show structural and functional damage, leading to a decrease in FAO. In other investigations, in which trophoblast cells were treated in vitro with FFA of different chain lengths, increasing LC-FA reduced LCHAD and CPT2 gene expression [51], increased the intracellular accumulation of lipid droplets, and caused histological mitochondrial damage and a loss of trophoblast invasiveness; whereas, SC-FA or MC-FA do not have the same significant effect. This mitochondrial structural injury is similar to that observed in the trophoblast of patients with EOPE or HELLP syndrome. However, no differences in CPT1 gene expression with FFA chain length have been found, with a similar histology to the control groups [52,53].

The complex involvement of lipid metabolism in the pathogenesis of pre-eclampsia goes beyond the study of FAO enzymes per se, as other metabolic pathways or proteins may be primarily affected by subsequent metabolic errors in FAO enzymes. Peroxisome proliferator-activated receptors (PPARs) are nuclear receptor proteins that regulate the gene expression involved in the regulation of lipid and glucose metabolism, inflammation, cell differentiation, and growth in multiple tissues. There are three isoforms (PPARα, PPARβ/δ, and PPARγ) activated by a wide variety of ligands, such as fatty acids and derivatives. They show distinct but overlapping expression and functions and are also expressed in the amnion, decidua, and villous placenta [54,55,56]. Most research focuses on the role of PPARγ in pre-eclampsia due to its involvement in lipid storage and syncytiotrophoblast differentiation, reporting reductions when compared to controls. Nevertheless, several study groups have published controversial results, rendering it impossible to draw conclusions at this time [57,58,59]. In pre-eclampsia, alterations in gene expression have also been demonstrated in other proteins that are involved in different areas of lipid metabolism, for example, in lipoprotein lipase (LPL), which hydrolyzes triglycerides into FFA to allow their transfer to the placenta, or in transport proteins, such as fatty acid transport proteins (FATPs), fatty acid translocase (FAT/CD36), and fatty acid binding proteins (FABPs), which enable LC-FA uptake into syncytiotrophoblast cells and their transfer from the maternal compartment to the fetal circulation [57,60,61].

In vitro studies in classical pre-eclampsia-like mouse models (after Nω-nitro-L-arginine-methyl ester injection, L-NAME) confirm a lower expression of LCHAD mRNA and proteins in the liver and placenta with respect to controls; however, other models, like the ApoC3-transgenic mice (suffering of an abnormal fatty acid metabolism and hypertriglyceridemia) or a mouse model with antiphospholipid syndrome (after β2-glycoprotein-I injection), present increased LCHAD expression [39,41]. This leads us to the hypothesis that FAO might play a different role in different factorial pre-eclampsia and might have a different cause-and-effect relationship.

A decrease in the gene expression of FAO enzymes in pre-eclampsia, especially on the fetal side of the placenta, leads to the lower utilization of FFA by the placenta with the accumulation of triglycerides on the fetal side, as previously reported [16]. Theoretically, the pathogenic sequence can start with decreased placental FAO, which would increase the FFA and triglyceride placental and maternal serum levels, leading to immune-resistance, lipotoxicity, and finally, endothelial damage. In favor of this hypothesis, we have shown in a previous paper that there is a positive correlation between FFA, triglycerides, and C-peptide with the anti-angiogenic factors of endothelial damage, such as the sFlt-1/PlGF ratio (soluble Fms-like Tyrosine Kinase-1 (sFlt-1)/placental growth factor (PlGF), and soluble endoglin (sEng) in maternal serum in pre-eclampsia [9]. The etiopathogenesis of LOPE has traditionally been related to metabolic syndrome and insulin resistance [62], which is closely linked to the accumulation of serum FFA. However, in our current report, we reveal that the decrease in FAO enzyme mRNA expression at the placenta also occurs in EOPE (in fact, no differences were found with respect to LOPE). This finding is also supported by our previous study, where higher FFA and C-peptide maternal levels were found in pre-eclampsia vs. controls, with no differences according to the time of onset of pre-eclampsia [9]. Although some metabolic dysfunctions might be independent of the time of onset of pre-eclampsia, the pathophysiological particularities that lead to each clinical spectrum are still unknown, indicating that other factors, such as genetic or immunological factors, must be involved [63,64].

### 4.3. Limitations of the Study

The sample size of each analyzed group was small, including singleton and twin pregnancies. The investigation of metabolomics, in contrast to genomics, transcriptomics, and proteomics, can more directly and accurately reflect the dynamic state of the organism. However, due to the specificity and the limited research previously described in the literature on this subject (with studies with an even smaller sample size than ours), this paper can serve as a basis for future investigation, in which our findings might be confirmed and related to other metabolic pathways, oxidative stress, inflammation, or even potential treatments.

## 5. Conclusions

The mRNA expression of the FAO enzymes LCHAD, MCAD, and CPT2 is lower in the placenta in pre-eclampsia, with more marked reductions observed in more severe cases and in those associated with EOPE or IUGR. These alterations are observed for both sides of the placenta, with more striking reductions on the fetal side.

## Figures and Tables

**Table 1:** Maternal, obstetric, and perinatal results.

MATERNAL RESULTS	Control *n* = 24	EOPE *n* = 12	LOPE *n* = 11	*p*-Value
Maternal age (years)	37.0 (7.0)	36.0 (16.0)	35.0 (3.0)	0.761
Ethnic Group:				0.352
Caucasian	18 [75.0%]	7 [58.3%]	7 [63.6%]	
Hispanic	6 [25.0%]	5 [41.7%]	3 [27.3%]	
Asian	0	0	1 [9.1%]	
Pregravid body mass index (kg/m^2^)	23.14 ± 2.73	22.05 ± 3.07	25.23 ± 3.73	0.636
Body mass index classification:				0.243
Underweight (<18.5)	0	1 [8.3%]	0	
Normal weight (18.5–24.9)	19 [79.2%]	6 [50.0%]	6 [54.5%]	
Overweight (25.0–29.9)	3 [12.5%]	2 [16.6%]	4 [36.4%]	
Obesity (>30.0)	0	0	1 [9.1%]	
Gestational weight gain (kg)	15.0 (9.0)	10.0 (6.0)	14.0 (8.0)	0.269
**OBSTETRIC RESULTS**				
Gestational age at study (weeks)	39.17 ± 1.12	31.75 ± 3.44	36.91 ± 1.22	*a* 1 × 10^−6^ * *b* 0.013 * *c* 1 × 10^−6^ *
Parity:				
Primigravid	6 [25.0%]	6 [50.0%]	6 [45.5%]	0.120
Multiparous	4 [16.6%]	3 [25.0%]	2 [18.2%]	0.380
Previous C-section	11 [45.8%]	3 [25.0%]	2 [18.2%]	0.419
Previous miscarriage	9 [37.5%]	2 [16.7%]	4 [36.6%]	0.333
Twin pregnancy	4 [16.7%]	2 [16.7%]	3 [27.4%]	0.612
Mode of pregnancy:				0.553
Spontaneous	19 [79.2%]	9 [75.0%]	9 [81.8%]	
ART (IUI)	0	1 [8.3%]	0	
ART (IVF)	5 [20.8%]	2 [16.7%]	2 [18.2%]	
Obstetric reason for cesarean section:				
A.—Fetal:				
Breech or transverse fetal position	7 [29.3%]	1 [8.3%]	1 [9.1%]	
Suspicion of fetal macrosomia	1 [4.1%]	0	0	
IUGR with critical Doppler	0	1 [8.3%]	1 [9.1%]	
Intrapartum fetal distress	0	2 [16.7%]	1 [9.1%]	
B.—Maternal:				
Severe pre-eclampsia	0	8 [66.7%]	5 [45.4%]	
HELLP syndrome (complete or partial)	0	4 [33.3%]	1 [9.1%]	
Iterative C-section or Previous C-section + Bishop test ≤6	10 [41.7%]	0	0	
Twin pregnancy + Bishop test ≤6	4 [16.7%]	0	1 [9.1%]	
Labor dystocia	0	0	1 [9.1%]	
Elective (advanced maternal age)	1 [4.1%]	0	0	
Fracture of femur head	1 [4.1%]	0	0	
Myopia magna	0	0	1 [9.1%]	
**PERINATAL RESULTS**	*n* = 28	*n* = 14	*n* = 14	
EFW (g)	3081.4 ± 578.9	1284.9 ± 473.8	2345.83 ± 471.9	*a* 1 × 10^−6^ * b 1.2 × 10^−4^ * *c* 4 × 10^−5^ *
Centile EFW	65.0 (63.0)	4.0 (7.0)	70.0 (70.0)	*a* 5 × 10^−5^ * b 0.719 *c* 0.001 *
Fetal growth restriction:				
SGA	1 [43.6%]	3 [21.4%]	0	*a* 0.022 * *b* 0.252 *c* 0.024 *
IUGR	0	8 [57.1%]	3 [21.4%]	*a* 2 × 10^−4^ * *b* 0.001 * c 5 × 10^−4^ *
Neonatal birth weight (g)	3320.0 (600.0)	1600.0 (540.0)	2440.0 (1045.0)	*a* 2 × 10^−6^ * *b* 0.019 * *c* 0.029 *
Neonatal birth weight centile	66.0 (32.0)	3.0 (5.0)	51.0 (92.0)	*a* 1 × 10^−6^ * *b* 0.719 *c* 0.001 *
Neonatal sex:				0.358
Male	12 [42.9%]	3 [21.4%]	6 [42.9%]	
Female	16 [57.1%]	11 [78.6%]	8 [57.1%]	
Umbilical artery pH at birth	7.30 ± 0.06	7.28 ± 0.07	7.28 ± 0.07	0.500
Placental weight	581.68 ± 130.85	319.49 ± 116.64	487.26 ± 136.21	*a* 3 × 10^−6^ * *b* 0.122 *c* 0.101

*Key:* EOPE: early-onset pre-eclampsia; LOPE: late-onset pre-eclampsia; C-section: cesarean section; ART: assisted reproductive technology; IUI: intrauterine insemination; IVF: in vitro fertilization; HELLP: hemolysis; elevated liver enzymes; low platelets; EFW: estimated fetal weight; SGA: small for gestational age; IUGR: intrauterine growth restriction. Qualitative variables are expressed as absolute values and percentages; *n* [%]. Quantitative variables are shown as mean ± standard deviation or as the median and interquartile range (in brackets) according to the distribution. *p*-value between the following groups: *a:* controls vs. EOPE; *b:* controls vs. LOPE; *c:* EOPE vs. LOPE. * There were significant differences among the groups (*p* < 0.05).

## Data Availability

Not applicable.

## Supplementary Materials

The following supporting information can be downloaded at: https://www.mdpi.com/article/10.3390/nu15183877/s1. Table S1: qPCR primers and conditions. Table S2: Relative LCHAD mRNA expression (a.u.). Table S3: Relative MCAD mRNA expression (a.u.). Table S4: Relative CPT1A mRNA expression (a.u.). Table S5: Relative CPT2 mRNA expression (a.u.).

## Abbreviations

AFLP	Acute fatty liver of pregnancy
a.u.	Arbitrary units
cDNA	Complementary DNA
COX-2	Cyclooxygenase-2
CPT	Carnitine palmitoyltransferase
Cq	Quantification cycle
EOPE	Early-onset pre-eclampsia
FA	Fatty acid
FABPs	Fatty acid-binding proteins
FAO	Fatty acid oxidation
FAT/CD36	Fatty acid translocase
FATPs	Fatty acid transport proteins
FFA	Free fatty acids
GAPDH	Glyceraldehyde-3-phosphate dehydrogenase
HDL	High-density lipoprotein
HELLP	Hemolysis, elevated liver function, and low platelets
IUGR	Intrauterine growth restriction
LC	Long chain
LCHAD	Long-chain 3-hydroxyacyl-CoA dehydrogenase
LDL	Low-density lipoprotein
L-NAME	Nω-nitro-L-arginine-methyl ester
LOPE	Late-onset pre-eclampsia
LPL	Lipoprotein lipase
MAPK	Mitogen-Activated Protein Kinases
MC	Medium chain
MCAD	Medium-chain acyl-CoA dehydrogenase
MPE	Mild pre-eclampsia
mRNA	Messenger RNA
NADPH	Nicotinamide adenine dinucleotide phosphate
NF-κB	Nuclear factor-κB
NIUGR	Not intrauterine growth restriction
PlGF	Placental growth factor
PPARs	Peroxisome proliferator-activated receptors
PUFA	Polyunsaturated fatty acids
RPL30	Ribosomal protein L30
RT-qPCR	Real-time quantitative PCR
SC	Short-chain
SCHAD	Short-chain 3-hydroxyacyl-CoA dehydrogenase
sEng	Soluble endoglin
sFlt-1	Soluble fms-like tyrosine kinase-1
SPE	Severe pre-eclampsia
VLCAD	Very-long-chain acyl-coA dehydrogenase
