# Supplementary material for: Decreased Fatty Acid Oxidation Gene Expression in Pre-Eclampsia According to the Onset and Presence of Intrauterine Growth Restriction
Source: Nutrients. 2023 Sep 6;15(18):3877. doi: 10.3390/nu15183877 (PMC10536348; doi:10.3390/nu15183877)
Supplement: Supplementary file 1 [file nutrients-15-03877-s001.zip › nutrients-2570130-supplementary.pdf]

nutrients

MDPI

Article

# Decreased Fatty Acid Oxidation Gene Expression in Preeclampsia according to the Onset and the Presence of Intrauterine Growth Restriction

**Alejandra Abascal-Saiz [1,†], Eva Fuente-Luelmo [2,†], María Haro [2], Victoria Fioravantti [3], Eugenia Antolín [1], María P. Ramos-Álvarez [2], and José L. Bartha [1,*]**

## SUPPLEMENTARY FILES

**Supplementary Table S1:** qPCR primers and conditions.

| | Product length (pb) | Primer Forward / Primer Reverse | Annealing temperature | Efficiency (%) | $R^2$ | Slope | Intercept |
|---|---|---|---|---|---|---|---|
| GAPDH | 87 | TGCACCACCAACTGCTTAGC / GGCATGGACTGTGGTCATGAG | 61 | 97.6 | 0.999 | -3.381 | 25.18 |
| LCHAD (HADHA) | 195 | ATCAAATCAGAAGTGCCGTCCT / CTCCCAGGCAGGATCCATTG | 62 | 96.1 | 0.988 | -3.418 | 47.426 |
| MCAD (ACADM) | 146 | ACCAAGTATGCCCTGGAAAGG / CGACCAGAATCAACCTCCCAA | 62 | 99.8 | 0.986 | -3.327 | 49.383 |
| RPL30 | 149 | AAAGCTGGGCAGTTGTTAGC / AAGACGAAAAAGTCGCTGGA | 60 | 91.2 | 0.991 | -3.553 | 42.151 |
| CPT1A | 288 | TTCAGTTCACGGTCACTCCG / TGACCACGTTCTTCGTCTGG | 61 | 93.9 | 0.953 | -3.477 | 51.124 |
| CPT2 | 129 | TTGAAGACACCATTAGGAGATACC / CCAGCTGCTCATGCAGTTCTTTT | 60 | 91.1 | 0.991 | -3.555 | 51.064 |

Key: qPCR: quantitative PCR, GAPDH: Glyceraldehyde-3-phosphate dehydrogenase, LCHAD: long-chain 3-hydroxyacyl-CoA dehydrogenase, HADHA: Hydroxyacyl-CoA dehydrogenase trifunctional multienzyme complex subunit alpha. MCAD / ACADM: Medium-chain acyl-CoA dehydrogenase, RPL30: Ribosomal protein L30, CPT: Carnitine palmitoyltransferase.

**Supplementary Table S2:** Relative LCHAD mRNA expression (a.u.)

| LCHAD | | | | | | | | | | |
|---|---|---|---|---|---|---|---|---|---|---|
| | *Comparison of different groups* | | | | | | | | | *Comparison of placenta sides* |
| | **Global** | | | **Maternal side** | | | **Fetal side** | | | |
| | | ***p* value: control vs. subgroup** | ***p* value: intra subgroup** | | ***p* value: control vs. subgroup** | ***p* value: intra subgroup** | | ***p* value: control vs. subgroup** | ***p* value: intra subgroup** | ***p* value** |
| Control, n=28 | 1.003 (0.383) | - | - | 0.908 (0.598) | - | - | 1.067 (0.584) | - | - | *0.733* |
| Preeclampsia, n=28 | 0.635 (0.276) | *$1.1 \times 10^{-5}$** | - | 0.636 (0.532) | *$5.6 \times 10^{-4}$** | - | 0.608 (0.250) | *$5.5 \times 10^{-4}$** | - | *0.677* |
| EOPE, n=14 | 0.625 (0.263) | *$3.3 \times 10^{-4}$** | *0.575* | 0.635 (0.440) | *0.005** | *0.512* | 0.583 (0.252) | *0.019** | *0.924* | *0.534* |
| LOPE, n=14 | 0.655 (0.318) | *0.003** | | 0.681 (0.766) | *0.04** | | 0.633 (0.334) | *0.013** | | *0.925* |
| SPE, n=14 | 0.635 (0.271) | *0.001** | *0.822* | 0.721 (0.497) | *0.017** | *0.987* | 0.648 (0.242) | *0.018** | *0.998* | *0.272* |
| MPE, n=14 | 0.635 (0.319) | *0.001** | | 0.574 (0.684) | *0.013** | | 0.608 (0.419) | *0.014** | | *0.650* |
| IUGR, n=10 | 0.635 (0.263) | *0.001** | *0.813* | 0.636 (0.608) | *0.017** | *0.726* | 0.583 (0.227) | *0.028** | *0.868* | *0.678* |
| NIUGR, n=17 | 0.635 (0.322) | *0.001** | | 0.681 (0.514) | *0.013** | | 0.681 (0.297) | *0.009** | | *0.959* |

*Key:* a.u.: arbitrary units, LCHAD: long-chain 3-hydroxyacyl-CoA dehydrogenase, EOPE: early-onset preeclampsia, LOPE: late-onset preeclampsia, SPE: severe preeclampsia, MPE: mild preeclampsia, IUGR: intrauterine growth restriction, NIUGR: Not intrauterine growth restriction. The results are expressed as the median and interquartile range (in brackets). *There were significant differences among the groups ($p < 0.05$).

**Supplementary Table S3:** Relative MCAD mRNA expression (a.u.)

| MCAD | | | | | | | | | | |
|---|---|---|---|---|---|---|---|---|---|---|
| | ***Comparison of different groups*** | | | | | | | | | ***Comparison of placenta sides*** |
| | **Global** | | | **Maternal side** | | | **Fetal side** | | | |
| | | ***p* value: control vs. subgroup** | ***p* value: intra subgroup** | | ***p* value: control vs. subgroup** | ***p* value: intra subgroup** | | ***p* value: control vs. subgroup** | ***p* value: intra subgroup** | ***p* value** |
| Control, n=28 | 1.020 ± 0.210 | - | - | 1.001 ± 0.235 | - | - | 1.058 ± 0.286 | - | - | *0.361* |
| Preeclampsia, n=28 | 0.755 + 0.247 | *7.67 ×10$^{-5}$** | - | 0.796 ± 0.334 | *0.011** | - | 0.719 ± 0.193 | *8 ×10$^{-6}$** | - | *0.172* |
| EOPE, n=14 | 0.708 ± 0.325 | *4.9 ×10$^{-4}$** | *0.571* | 0.734 ± 0.409 | *0.021** | *0.537* | 0.669 ± 0.233 | *1.6 ×10$^{-4}$** | *0.641* | *0.512* |
| LOPE, n=14 | 0.798 ± 0.143 | *0.013** | | 0.852 ± 0.247 | *0.265* | | 0.759 ± 0.152 | *0.02** | | *0.244* |
| SPE, n=14 | 0.685 ± 0.286 | *1.6 ×10$^{-4}$** | *0.284* | 0.754 ± 0.398 | *0.037** | *0.753* | 0.650 ± 0.217 | *4.1 ×10$^{-5}$** | *0.374* | *0.151* |
| MPE, n=14 | 0.819 ± 0.194 | *0.024** | | 0.834 ± 0.270 | *0.193* | | 0.783 ± 0.150 | *0.004** | | *0.709* |
| IUGR, n=10 | 0.744 ± 0.344 | *0.004** | *0.979* | 0.780 ± 0.464 | *0.155* | *0.998* | 0.659 ± 0.269 | *3.1 ×10$^{-4}$** | *0.634* | *0.371* |
| NIUGR, n=17 | 0.762 ± 0.165 | *0.002** | | 0.793 ± 0.245 | *0.061* | | 0.753 ± 0.133 | *1 ×10$^{-4}$** | | *0.334* |

*Key*: a.u.: arbitrary units, MCAD: medium-chain acyl-CoA dehydrogenase, EOPE: early-onset preeclampsia, LOPE: late-onset preeclampsia, SPE: severe preeclampsia, MPE: mild preeclampsia, IUGR: intrauterine growth restriction, NIUGR: Not intrauterine growth restriction. The results are expressed as the mean ± standard deviation. *There were significant differences among the groups ($p < 0.05$).

**Supplementary Table S4:** Relative CPT1A mRNA expression (a.u.)

| CPT1A | | | | | | | | | | |
|---|---|---|---|---|---|---|---|---|---|---|
| | ***Comparison of different groups*** | | | | | | | | | ***Comparison of placenta sides*** |
| | **Global** | | | **Maternal side** | | | **Fetal side** | | | |
| | | ***p* value: control vs. subgroup** | ***p* value: intrasubgroup** | | ***p* value: control vs. subgroup** | ***p* value: intrasubgroup** | | ***p* value: control vs. subgroup** | ***p* value: intrasubgroup** | ***p* value** |
| Control, n=28 | 1.330 ± 0.760 | - | - | 1.346 ± 0.643 | - | - | 1.251 ± 0.722 | - | | *0.526* |
| Preeclampsia, n=28 | 1.379 ± 0.798 | *0.818* | - | 1.382 ± 0.823 | *0.859* | - | 1.293 ± 0.615 | *0.822* | - | *0.623* |
| EOPE, n=14 | 1.629 ± 0.617 | *0.482* | *0.241* | 1.508 ± 0.700 | *0.793* | *0.672* | 1.475 ± 0.517 | *0.619* | *0.456* | *0.884* |
| LOPE, n=14 | 1.147 ± 0.895 | *0.476* | | 1.265 ± 0.934 | *0.940* | | 1.150 ± 0.665 | *0.889* | | *0.353* |
| SPE, n=14 | 1.496 ± 0.751 | *0.804* | *0.735* | 1.551 ± 0.734 | *0.687* | *0.488* | 1.411 ± 0.702 | *0.772* | *0.680* | *0.582* |
| MPE, n=14 | 1.270 ± 0.852 | *0.970* | | 1.225 ± 0.896 | *0.870* | | 1.184 ± 0.527 | *0.953* | | *0.528* |
| IUGR, n=10 | 1.197 ± 0.694 | *0.880* | *0.575* | 1.290 ± 0.744 | *0.975* | *0.856* | 1.217 ± 0.600 | *0.990* | *0.892* | *0.997* |
| NIUGR, n=17 | 1.504 ± 0.861 | *0.757* | | 1.445 ± 0.892 | *0.906* | | 1.343 ± 0.640 | *0.905* | | *0.306* |

*Key*: a.u.: arbitrary units, CPT: carnitine palmitoyltransferase, EOPE: early-onset preeclampsia, LOPE: late-onset preeclampsia, SPE: severe preeclampsia, MPE: mild preeclampsia, IUGR: intrauterine growth restriction, NIUGR: Not intrauterine growth restriction. The results are expressed as the mean ± standard deviation. *There were significant differences among the groups ($p < 0.05$).

**Supplementary Table S5:** Relative CPT2 mRNA expression (a.u.)

| CPT2 | | | | | | | | | | |
|---|---|---|---|---|---|---|---|---|---|---|
| | *Comparison of different groups* | | | | | | | | | *Comparison of placenta sides* |
| | **Global** | | | **Maternal side** | | | **Fetal side** | | | |
| | | ***p* value: control vs. subgroup** | ***p* value: intrasubgroup** | | ***p* value: control vs. subgroup** | ***p* value: intrasubgroup** | | ***p* value: control vs. subgroup** | ***p* value: intrasubgroup** | ***p* value** |
| Control, n=28 | 1.028 ± 0.247 | - | - | 1.051 ± 0.354 | - | | 1.175 ± 0.332 | - | - | *0.139* |
| Preeclampsia, n=28 | 0.745 ± 0.250 | *1.1 ×10⁻⁴** | | 0.782 ± 0.335 | *0.006** | - | 0.854 ± 0.301 | *5.7 ×10⁻⁴** | - | *0.391* |
| EOPE, n=14 | 0.725 ± 0.166 | *0.002** | *0.908* | 0.753 ± 0.312 | *0.036** | *0.899* | 0.841 ± 0.198 | *0.014** | *0.983* | *0.456* |
| LOPE, n=14 | 0.766 ± 0.319 | *0.008** | | 0.813 ± 0.365 | *0.113* | | 0.864 ± 0.370 | *0.013** | | *0.687* |
| SPE, n=14 | 0.690 ± 0.172 | *4.8 ×10⁻⁴** | *0.494* | 0.687 ± 0.292 | *0.007** | *0.336* | 0.849 ± 0.237 | *0.013** | *0.997* | *0.205* |
| MPE, n=14 | 0.801 ± 0.307 | *0.023** | | 0.878 ± 0.359 | *0.297* | | 0.858 ± 0.360 | *0.013** | | *0.801* |
| IUGR, n=10 | 0.655 ± 0.247 | *2.4 ×10⁻⁴** | *0.251* | 0.652 ± 0.352 | *0.005** | *0.224* | 0.803 ± 0.245 | *0.007** | *0.796* | *0.271* |
| NIUGR, n=17 | 0.812 ± 0.274 | *0.021** | | 1.051 ± 0.354 | *0.259* | | 0.888 ± 0.337 | *0.019** | | *0.880* |

*Key:* a.u.: arbitrary units, CPT: carnitine palmitoyltransferase, EOPE: early-onset preeclampsia, LOPE: late-onset preeclampsia, SPE: severe preeclampsia, MPE: mild preeclampsia, IUGR: intrauterine growth restriction, NIUGR: Not intrauterine growth restriction. The results are expressed as the mean ± standard deviation. *There were significant differences among the groups ($p < 0.05$).
